# Supplementary material for: The Systems Biology Research Tool: evolvable open-source software
Source: BMC Syst Biol. 2008 Jun 29;2:55. doi: 10.1186/1752-0509-2-55 (PMC2446383; doi:10.1186/1752-0509-2-55)
Supplement: Additional file 1 — SBRT Archive. An archive of the current version of the Systems Biology Research Tool. [file 1752-0509-2-55-S1.zip › sbrt-1.4.0/doc/developers_guide/api/sbrt/shell/proc/fba/SbmlWriter.html]

SbmlWriter


|  |  |  |
| --- | --- | --- |
| **Overview** \| **Package** \| **Class** \| **Use** \| **Tree** \| **Deprecated** \| **Index** \| **Help** | |  |
| **PREV CLASS**   **NEXT CLASS** | **FRAMES**    **NO FRAMES**     **All Classes** |  |
| SUMMARY: NESTED \| FIELD \| CONSTR \| METHOD | DETAIL: FIELD \| CONSTR \| METHOD |  |


---


## sbrt.shell.proc.fba Class SbmlWriter

```
java.lang.Object
  sbrt.shell.proc.fba.SbmlWriter
```

---

``` public class SbmlWriter extends java.lang.Object ```

This class is used to write fluxomes in SBML format.

---

**Constructor Summary**

`SbmlWriter()`


| **Method Summary** | |
| --- | --- |
| `void` | `write(IrrevRxnFluxome fluxome, java.lang.String sbmlFileName)`             Writes the provided fluxome in SBML format to a file with the provided name. |

**Methods inherited from class java.lang.Object**

`clone, equals, finalize, getClass, hashCode, notify, notifyAll, toString, wait, wait, wait`

**Constructor Detail**

### SbmlWriter

```
public SbmlWriter()
```


**Method Detail**

### write

```
public void write(IrrevRxnFluxome fluxome,
                  java.lang.String sbmlFileName)
```

:   Writes the provided fluxome in SBML format to a file with the provided name.

    :   **Parameters:**: `fluxome` - the fluxome to be written in SBML format.: `sbmlFileName` - the name of the SBML file to be created.


---


|  |  |  |
| --- | --- | --- |
| **Overview** \| **Package** \| **Class** \| **Use** \| **Tree** \| **Deprecated** \| **Index** \| **Help** | |  |
| **PREV CLASS**   **NEXT CLASS** | **FRAMES**    **NO FRAMES**     **All Classes** |  |
| SUMMARY: NESTED \| FIELD \| CONSTR \| METHOD | DETAIL: FIELD \| CONSTR \| METHOD |  |


---
